# *Campylobacter jejuni* uses energy taxis and a dehydrogenase enzyme for l-fucose chemotaxis

**DOI:** 10.1128/mbio.02732-23

**Published:** 2023-11-30

**Authors:** Bibi Zhou, Jolene M. Garber, Jiri Vlach, Parastoo Azadi, Kenneth K. S. Ng, Jorge C. Escalante-Semerena, Christine M. Szymanski

**Affiliations:** 1 Department of Microbiology, University of Georgia, Athens, Georgia, USA; 2 Complex Carbohydrate Research Center, University of Georgia, Athens, Georgia, USA; 3 Department of Chemistry and Biochemistry, University of Windsor, Windsor, Ontario, Canada; Emory University School of Medicine, Atlanta, Georgia, USA

**Keywords:** *Campylobacter jejuni*, l-fucose, energy taxis, chemotaxis

## Abstract

*Campylobacter jejuni* is a leading cause of bacterial diarrhea worldwide, and infection in infants is associated with growth stunting in low- and middle-income countries. Approximately half of all *C. jejuni* isolates are asaccharolytic, while the remainder encode enzymes capable of l-fucose catabolism. Our previous study suggested that breastfed infants originating from sub-Saharan Africa and South Asia were colonized less frequently with l-fucose-metabolizing *C. jejuni* isolates compared to asaccharolytic strains. We also showed that FucX is an l-fucose dehydrogenase that binds to its NADP^+^ cofactor even in the absence of l-fucose and that this enzyme is sufficient for l-fucose chemotaxis when introduced into strains lacking the metabolic pathway. This study indicates that FucX may influence l-fucose chemotaxis by increasing cellular NADPH/NADP^+^ ratios, which subsequently affect the energy taxis components, CetABC that respond to shifting gradients of electron acceptors and donors. Furthermore, *C. jejuni* CetAB and CetAC can complement *Escherichia coli* aerotaxis. Our data support a model in which the shift in NADP^+^ levels, impacted by the FucX dehydrogenase, affects chemotaxis in response to l-fucose. This swimming behavior can be phenocopied with a homologous l-fucose dehydrogenase from *Burkholderia multivorans*. Taken together, our work provides a possible explanation for why l-fucose-metabolizing *C. jejuni* isolates swim away from intestinal epithelial cells toward free fucose in the lumen where they are subsequently cleared by breastfed infants.

**IMPORTANCE:**

In this study, we identify a separate role for the *Campylobacter jejuni* l-fucose dehydrogenase in l-fucose chemotaxis and demonstrate that this mechanism is not only limited to *C. jejuni* but is also present in *Burkholderia multivorans*. We now hypothesize that l-fucose energy taxis may contribute to the reduction of l-fucose-metabolizing strains of *C. jejuni* from the gastrointestinal tract of breastfed infants, selecting for isolates with increased colonization potential.

## INTRODUCTION

*Campylobacter jejuni* is a leading cause of bacterial gastrointestinal disease worldwide. Infection typically occurs through the consumption of contaminated poultry products, milk, or water. Although patients usually show symptoms of self-limiting diarrhea, *C. jejuni* infection can lead to more serious sequelae including Guillain-Barré syndrome, reactive arthritis, environmental enteric dysfunction (EED), and irritable bowel syndrome (1, 2). EED is a subclinical chronic disorder that results from a combination of improper nutrient absorption, intestinal injury, and prolonged inflammation that leads not only to growth stunting but also impaired cognitive development, decreased oral vaccine responses, and higher risk of metabolic syndrome and its related cardiovascular sequelae (3). In addition, the World Health Organization (WHO) considers fluoroquinolone-resistant *Campylobacter* spp. as high priority pathogens for which development of new antibiotics requires urgent attention (4).

*C. jejuni* was once considered asaccharolytic, but we and others have shown that many *C. jejuni* isolates possess gene clusters (*cj0481-cj0490* in *C. jejuni* NCTC 11168) encoding functional enzymes for l-fucose metabolism (5, 6) and chemotaxis (7). These gene clusters are present as the *fuc* operon in over 60% of sequenced strains, and the expression of this operon is repressed by Cj0480c (fucose operon repressor/transcriptional regulator) when l-fucose is absent (5–7). We subsequently showed that d-arabinose is also metabolized by the enzymes in this pathway, also derepresses the operon, and contributes to microbial taxis (8).

In low- and middle-income countries, *C. jejuni* infection in infants has been associated with growth stunting and mortality (9, 10). Our research group also showed there are significantly higher proportions of *C. jejuni* in breastfed infants compared to non-breastfed infants that participated in the Global Enterics Multicenter Study (11). This relationship was also observed in breastfed infants in Ethiopia (2). Studies by our group and others showed that *Bacteroides vulgatus* and *Bacteriodes fragilis* promote *C. jejuni* growth in the presence of mucins by secreting fucosidases that release l-fucose from mucin (8, 12). Gut commensals such as *Bifidobacterium* spp. and *Bacteroides* spp. can also release l-fucose from fucosylated human milk oligosaccharides (HMOs) (13, 14). In breastfed infants, the amount of free l-fucose reaches up to 4–5 mg/g of fecal sample, which can potentially act as a nutrient source for l-fucose-metabolizing *C. jejuni* isolates (15). Interestingly, our group reported that in *Campylobacter-*infected infant feces, there is a significantly higher proportion of non-l-fucose-metabolizing *Campylobacter* than l-fucose-metabolizing *Campylobacter* in breastfed infants, while the ratios are nearly equal in non-breastfed infants (11).

We hypothesized that l-fucose chemotaxis plays a role in this process prompting l-fucose-metabolizing *C. jejuni* to swim toward free l-fucose in the intestinal lumen with subsequent expulsion through peristalsis into waste more quickly, while non-l-fucose-metabolizing *C. jejuni* swim toward the intestinal mucosa and cause disease, thus remaining in infants longer and growing to higher abundance by the time fecal samples were collected for the study. Organic acids, derived from the tricarboxylic acid cycle, and amino acids are preferred carbon sources for all *C. jejuni* strains, including l-fucose-metabolizing strains where fucose metabolism can be inhibited when excess l-serine, l-glutamic acid, and l-aspartic acid are added to the growth medium (8). However, in the gastrointestinal environment of breastfed infants, carbohydrates such as l-fucose are a rich source of nutrients, in comparison to adults, and l-fucose chemotaxis and utilization cannot be ignored.

Among the enzymes necessary for l-fucose metabolism, FucX (Cj0485) was the only member found to also play a key role in *C. jejuni* chemotaxis while *cj0481*, *cj0483*, *cj0484*, *cj0486*, *cj0487,* and *cj0488* mutants still showed l-fucose chemotaxis (7, 8). Notably, deletion of the l-fucose permease (*fucP, cj0486*) did not affect l-fucose chemotaxis, while the addition of *fucX* into the non-l-fucose-metabolizing *C. jejuni* strain 81–176 provided the microbe with the ability to chemotax to l-fucose. These results suggested that l-fucose metabolism is not required for *C. jejuni* chemotaxis, and FucX may play an indirect role in this process (7, 8). FucX is an l-fucose dehydrogenase that converts l-fucose and NADP^+^ into l-fuconolactone and NADPH (8). In our X-ray crystallography studies, we observed FucX bound to NADP^+^, even in the absence of l-fucose (8). We hypothesized that FucX controls l-fucose chemotaxis by altering the NADPH/NADP^+^ levels within the cell and influences the intracellular redox status in *C. jejuni* through the binding of NADP^+^.

Bacteria sense cellular redox changes by a process known as energy taxis (16) and the *Escherichia coli* energy taxis (also called aerotaxis) receptor Aer is the model for this system. Aer is a membrane-embedded protein with cytosolic Per-Arnt-Sim (PAS) and histidine kinase, adenylate cyclase, methyl-accepting protein, and phosphatase (HAMP) domains, in addition to a kinase control module (17). The two PAS domains within the dimer each bind flavin adenine dinucleotide (FAD) cofactors that sense the redox status of the cell. This sensing in turn alters the conformation of the associated CheA kinase, affecting amounts of phosphorylated CheY and triggering the regulation of flagellar rotational direction through conformational shifts in the protein following FAD reduction (18). In *C. jejuni*, CetA and CetB were reported to act as the bipartite energy taxis system (19, 20). CetB is predicted to be the PAS domain, while CetA possesses a predicted transmembrane region, the HAMP domain, and the C-terminal highly conserved signaling domain present in *E. coli* Aer (21). Later, it was demonstrated that *C. jejuni* also possesses a CetB homolog that was named CetC, which also plays a role in energy taxis (19, 20).

This work examines the effect of FucX in *C. jejuni* redox sensing and the contribution of the *C. jejuni* CetABC energy taxis system in l-fucose chemotaxis. Our previous research indicated that FabG is a FucX homolog in *Burkholderia multivorans* (22), and in this study, we show that FabG can phenocopy *C. jejuni* l-fucose chemotaxis suggesting that dehydrogenase-related chemotaxis systems might be more common than expected.

## RESULTS

### NADP^+^ binding with FucX may play a key role in l-fucose chemotaxis

**Fig 1:**
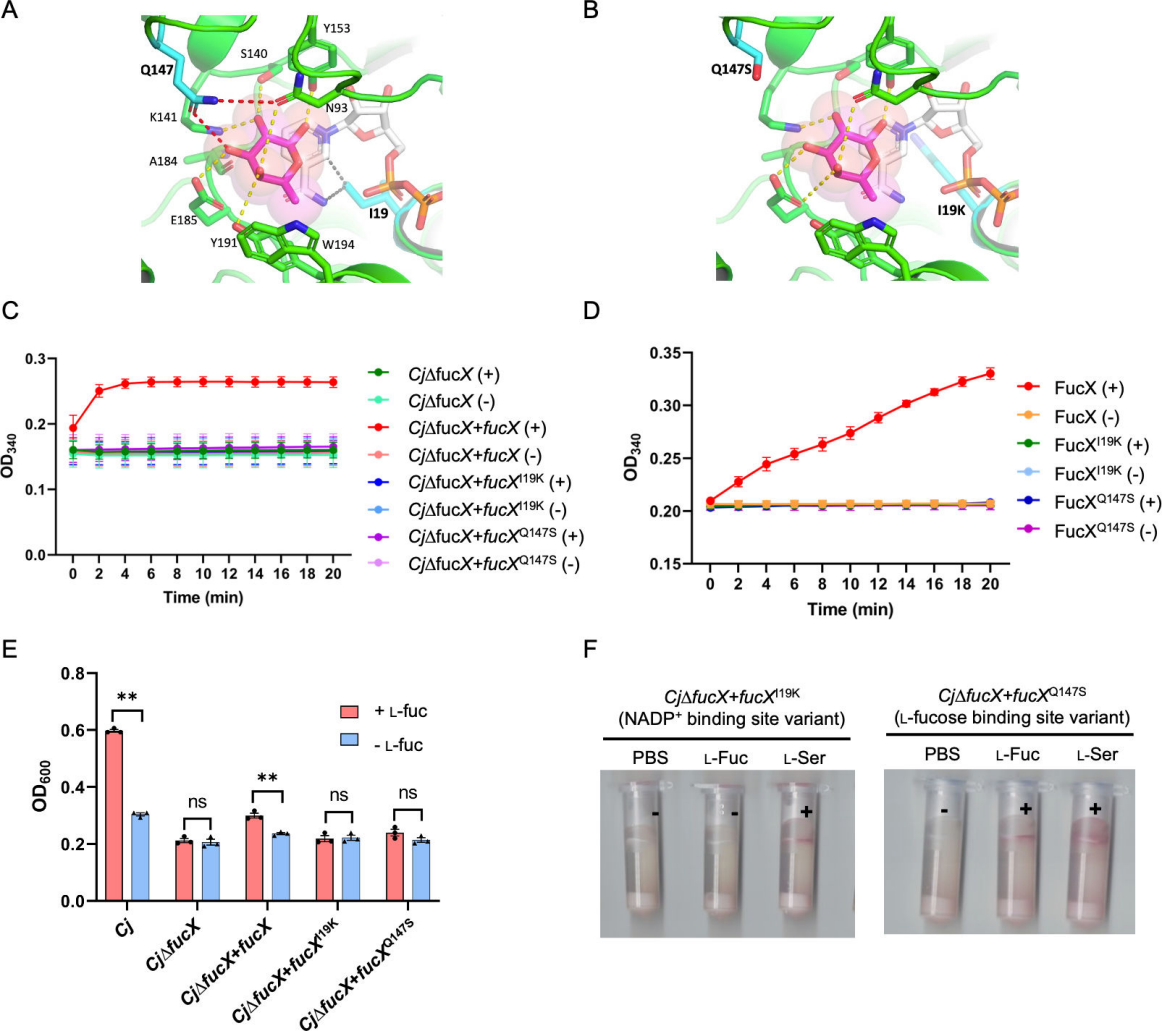
(**A**) Molecular model of *C. jejuni* FucX bound to l-fucose and NADP^+^. The predicted location of l-fucose was generated by superimposing the crystal structure of the ternary complex with *B. multivorans* FabG (4GVX) onto the crystal structure of the FucX binary complex with NADP^+^ (6DS1). The predicted hydrogen bonds that are lost in the Q147S mutant are shown as red dashed lines. The predicted van der Waals contacts that are lost in the I19K mutant are shown as dashed gray lines. Hydrogen bonds between l-fucose and FucX are shown as dashed yellow lines. l-Fucose is colored in magenta, and NADP^+^ is colored in white. (**B**) Molecular model of *C. jejuni* FucX showing stereochemically reasonable conformations for the Ser-147 and Lys-19 residues found in the Q147S and I19K mutants, respectively. (**C**) l-Fucose dehydrogenase activity of *C. jejuni* cell lysates from the indicated *fucX* mutant and complements: (+), with l-fucose; (−), no l-fucose. Data points are an average of three biological replicates. (**D**) l-Fucose dehydrogenase activity of indicated FucX pure proteins: (+), with l-fucose; (−), no l-fucose. Data points are an average of three biological replicates. (**E**) Growth of *C. jejuni fucX* mutants in minimum essential medium α supplemented with l-fucose, each data point represents one biological replicate. (**F**) Representative tube-based chemotaxis of *C. jejuni fucX* point mutants: (+), red, positive results; (−), no color, negative results; PBS, negative control; l-Fuc, l-fucose, test compound; l-Ser, l-serine, positive control. All assays were repeated at least three times. Error bars represent the standard error of the mean. **, significant, *P* ≤ 0.01; ns, not significant as determined by the Student’s *t* test.

To test our hypothesis that FucX binding to NADP^+^ affects l-fucose chemotaxis, we constructed an FucX NADP^+^-binding site variant (*C. jejuni*∆*fucX+fucX*^I19K^) and an FucX l-fucose-binding site variant (*C. jejuni*∆*fucX+fucX*^Q147S^), based on the predicted ligand (for l-fucose) and known ligand (for NADP^+^) binding sites in the FucX crystal structure (Fig. 1A), and the predicted interactions of mutated protein structures with their ligands are shown in Fig. 1B (8). Protein analyses followed by western blotting indicated FucX^I19K^ and FucX^Q147S^ show similar stability compared to the WT FucX at room temperature (RT; Fig. S1A and B). Dehydrogenase assays using whole-cell lysates and purified proteins showed that both mutants lost enzymatic activity (Fig. 1C and D). In the l-fucose growth assays, neither variant showed enhanced growth in the presence of l-fucose in minimal medium (Fig. 1E).

Chemotaxis assays with the variants showed that the FucX^I19K^ NADP^+^-binding site mutant completely lost l-fucose chemotaxis, which further supports the connection between NADPH/NADP^+^ and l-fucose chemotaxis (Fig. 1F). While the l-fucose chemotaxis results of the FucX^Q147S^ l-fucose-binding site variant yielded inconsistent results, although our controls performed appropriately in all repeats. So, we tested NADP^+^ binding to FucX^WT^ and its binding site variants using thermal shift assays to confirm the binding of NADP^+^ with FucX and its variants (Fig. S1C through E). A protein melting temperature (∆*T_m_*) change of more than 2°C is a strong indicator of ligand binding (23). The *T_m_* values of FucX, FucX^I19K^, and FucX^Q147S^ with and without NADP^+^ are shown in Table 1. The ∆*T_m_* of each protein indicates that all proteins show at least some binding to NADP^+^, but FucX^I19K^ showed the least change in thermostability compared with FucX and FucX^Q147S^. In the presence of NADP^+^, the *T_m_* value of FucX^I19K^ was approximately 4°C less than FucX^WT^ and FucX^Q147S^, suggesting that the Ile to Lys substitution decreases NADP^+^-binding affinity and FucX stability at higher temperatures. This result is consistent with the activity assays, growth curves, and chemotaxis assays for this mutant leading us to further explore whether NADP^+^ binding to FucX affects l-fucose chemotaxis through altering the ratio of free NADPH/NADP^+^ within the cell.

**TABLE 1:** ∆*T_m_* of FucX, FucX^I19K^, and FucX^Q147S^ with and without NADP^+^

Proteins	+ NADP^+^	− NADP^+^	∆*T_m_*^*a*^
FucX	58.4 ± 0.51°C	55.0 ± 0.00°C	3.4 ± 0.51°C
FucX^I19K^	54.6 ± 0.58°C	52.0 ± 0.00°C	2.6 ± 0.58°C
FucX^Q147S^	61.0 ± 0.00°C	54.0 ± 0.00°C	7.0 ± 0.00°C

^*a*^ Δ*T*_*m*_ is the result of subtracting the melting temperatures of each protein with and without the NADP^+^ ligand.

**Fig 2:**
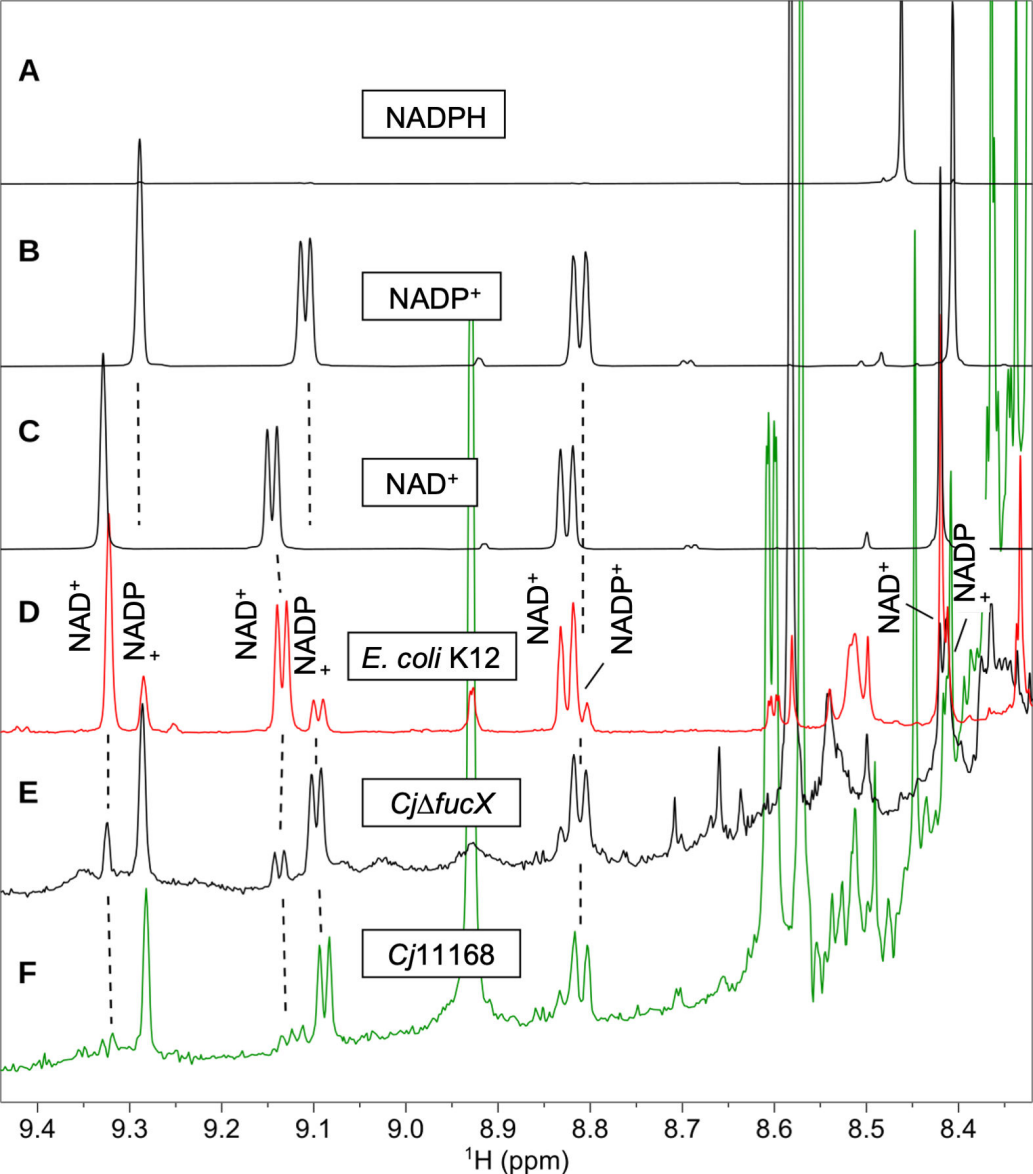
Partial representative ^1^H NMR spectra of NAD^+^ and NADP^+^ in *E. coli* K12 and *C. jejuni* (*Cj*) cell extracts. ^1^H NMR spectra of (**A**) NADPH, (**B**) NADP^+^, and (**C**) NAD^+^ standards acquired after eight scans. (**D**) ^1^H NMR spectrum of the *E. coli* K12 extract acquired after 1,880 scans (in red), and (**E**) ^1^H spectrum of the *C. jejuni* 11168*∆fucX* (*Cj∆fucX*) extract acquired after 15,280 scans. (**F**) ^1^H spectrum of the *C. jejuni* 11168 (*Cj*11168) extract acquired after 20,480 scans.

Our previous results showed that the preferred cofactor for FucX is NADP^+^ rather than NAD^+^ (8). NADH is the respiratory electron donor of complex I in *E. coli* (24), while in *Helicobacter pylori* and *C. jejuni*, NADPH and flavodoxin are the electron donors of complex I, respectively (25–27). To confirm that NADP^+^ exists in sufficient quantities in *C. jejuni*, we used NMR to visualize the levels of NAD^+^ and NADP^+^ in *E. coli* and *C. jejuni* and found that in *C. jejuni*, the quantity of NADP^+^ is much higher than NAD^+^, while in *E. coli*, the amount of NAD^+^ is higher than NADP^+^ (Fig. 2; Table S1), indicating that NADP is the dominant co-factor in *C. jejuni*.

We then calculated the ratio of NADPH/NADP^+^ in cell lysates of *C. jejuni*∆*fucX* and *C. jejuni*∆*fucX + fucX* using the Promega NADP^+^/NADPH Measurement Kit. However, we did not observe significant differences between these two strains (Fig. S1F). We speculated that other cofactors were being reduced by the imbalanced NADPH levels, hindering our efforts to measure a direct impact using the kit, and we demonstrated this was indeed the case once we determined that quick FAD reduction could be involved (see results below).

### NADPH/NADP^+^ ratio changes are important in l-fucose chemotaxis in *C. jejuni*

**Fig 3:**
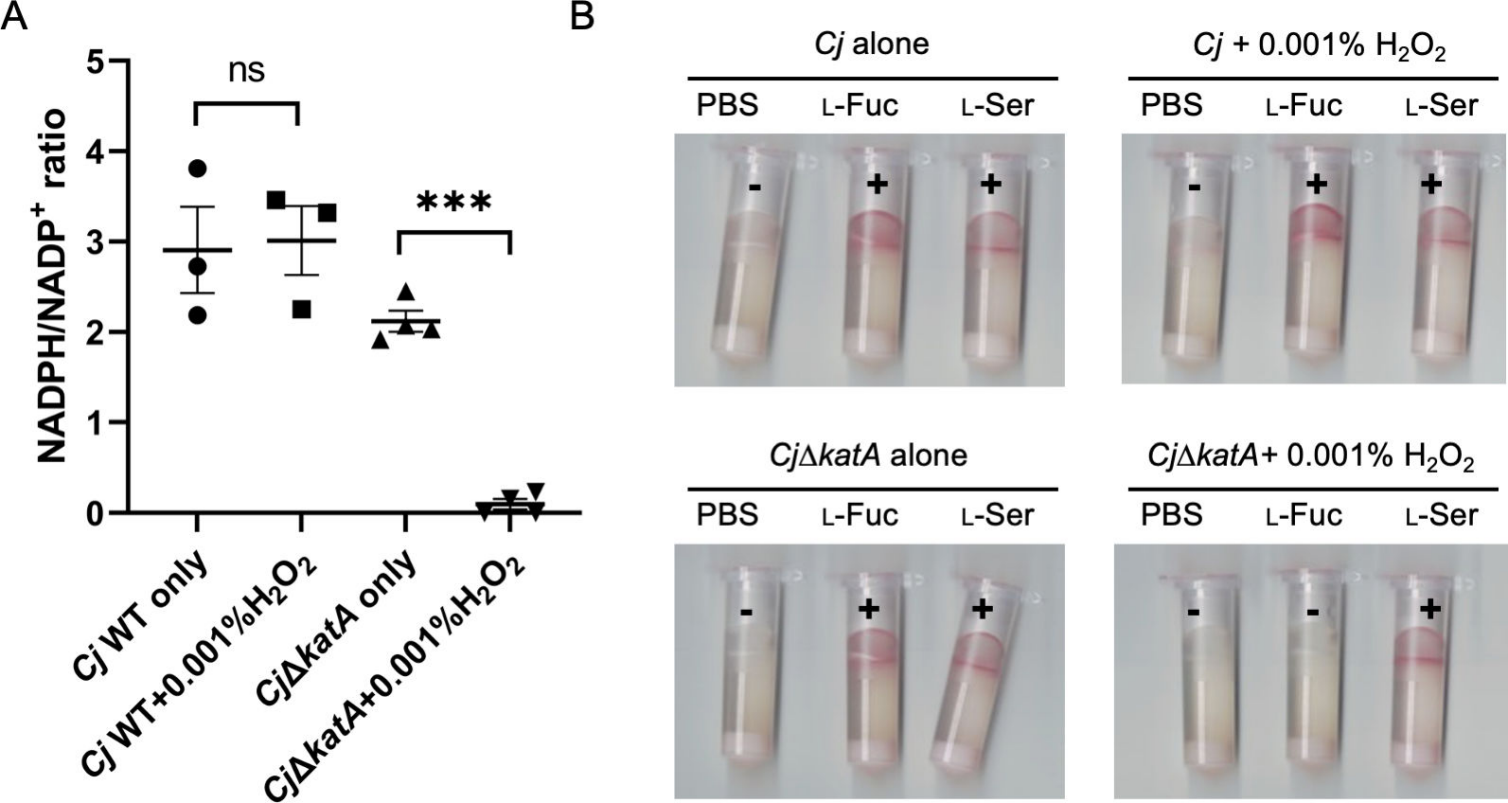
(**A**) NADPH/NADP^+^ ratio of *C. jejuni* (*Cj*) WT and *C. jejuni∆katA* treated with or without H_2_O_2_ using the Promega NADPH/NADP^+^ Measurement Kit. (**B**) Representative tube-based chemotaxis assays of *C. jejuni* WT and *C. jejuni*∆*katA* treated with and without H_2_O_2_. (+), red, positive results; (−), no color, negative results. PBS, negative control; l-Fuc, l-fucose, test compound; l-Ser, l-serine, positive control. All assays were repeated at least three times. Each data point represents one biological replicate. Error bars represent standard errors of the mean. ***, *P* ≤ 0.001; ns, not significant as determined by the Student’s *t* test.

Since we were unable to detect differences in the ratio of NADPH/NADP^+^ in *C. jejuni*∆*fucX* and *C. jejuni*∆*fucX + fucX* using the Promega NADPH/NADP^+^ Measurement Kit, we changed our strategy toward assessing the impact of altering NADPH/NADP^+^ ratios on l-fucose chemotaxis in a fixed system. To do this, we treated the *C. jejuni* catalase mutant, ∆*katA*, with H_2_O_2_. Catalase is the primary enzyme necessary for the breakdown of H_2_O_2_ into H_2_O and O_2_ (28) . Thus, treating *C. jejuni*∆*katA* with H_2_O_2_ forces the microbe to use NADPH-dependent secondary pathways to breakdown H_2_O_2_, causing the consumption of NADPH and generating more NADP^+^ (29). After treating *C. jejuni*∆*katA* with H_2_O_2_, we detected only minor levels of NADPH, while NADPH levels in untreated *C. jejuni*∆*katA* are comparable with *C. jejuni* WT (Fig. 3A). This suggests that H_2_O_2_ does not affect the NADPH/NADP^+^ ratios in WT and confirms that H_2_O_2_ decreases NADPH in the *C. jejuni*∆*katA* background. We then tested the chemotaxis ability of *C. jejuni*∆*katA* in the presence of H_2_O_2_ and observed that l-fucose chemotaxis was inhibited, while neither *C. jejuni*∆*katA* alone nor *C. jejuni* WT treated with H_2_O_2_ influenced l-fucose chemotaxis (Fig. 3B).

Overall, the results suggest that alterations in the NADPH/NADP^+^ ratio affect l-fucose chemotaxis. We thus hypothesized that l-fucose chemotaxis may be caused by NADP^+^ binding to FucX which affects NADPH/NADP^+^ in the cell.

### CetA, CetB, and CetC play roles in l-fucose energy taxis

*C. jejuni* strain 11168 has 10 transducer-like proteins (Tlps), Tlp1-10 (30). Recent studies suggested that Tlp2, Tlp3, Tlp4, and Tlp10 are l-fucose chemoreceptors in *C. jejuni* 11168 (31, 32). Based on these results, we constructed several of the mutants, and the chemotaxis comparisons indicated that they remain individually capable of l-fucose chemotaxis under our assay conditions (Fig. S2)

**Fig 4:**
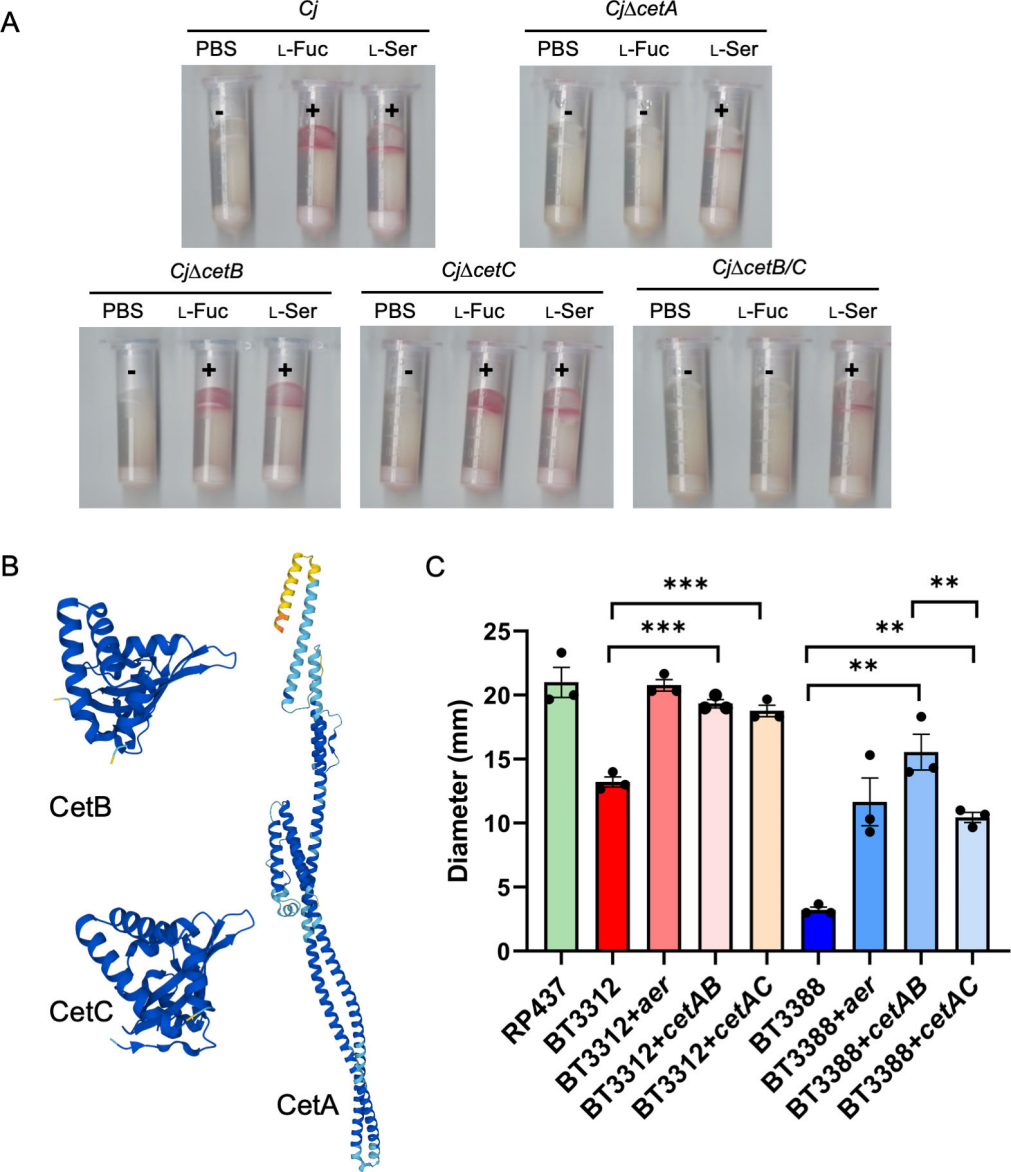
(**A**) Representative tube-based chemotaxis of *C. jejuni tlp* mutants. (+), red, positive results; (−), no color, negative results; PBS, negative control; l-Fuc, l-fucose, test compound; l-Ser, l-serine, positive control. (**B**) Predicted protein structure of CetA, CetB, and CetC by Alphafold2. (**C**) Aerotaxis halo sizes of *E. coli* taxis receptor mutants complemented with *C. jejuni* CetAB or CetAC. RP437, parental strain of BT3312 and BT3388; genotype, F-, *thr-1, araC14, leuB6(Am), fhuA31, lacY1, tsx-78, λ-, eda-50, hisG4(Oc), rfbC1, rpsL136(strR), xylA5, mtl-1, metF159(Am), thiE1*; BT3312, *E. coli* RP437 ∆*aer-1* ∆*tsr-7021*; BT3388, *E. coli* RP437 ∆*aer::erm* ∆*tsr-7021* ∆*tar-tap-5201 trg*::Tn*10*. All assays were repeated at least three times. Each data point represents one biological replicate. Error bars represent standard errors of the mean. ***, *P* ≤ 0.001; **, *P* ≤ 0.01 as determined by the Student’s *t* test.

We thus constructed other *tlp* mutants associated with energy taxis (*cet* mutants) in *C. jejuni* and performed our chemotaxis assay on each one. We observed loss of l-fucose chemotaxis in *C. jejuni*∆*tlp9* (Fig. 4A), while the other mutants all showed movement to l-fucose (Fig. S2). Previous research showed that Tlp9 (Cj1190c, also known as CetA) and Cj1189c (also known as CetB) form a bipartite Aer-like redox sensor (20). CetB is predicted to be a separate PAS-domain sensory protein which detects the energy/redox status of the cell, while CetA is predicted to act as a transducer protein, containing both the HAMP and kinase control domains and sequentially triggering flagellar rotation. The Alphafold2-predicted protein structures of CetA and CetB are shown in Fig. 4B (20, 33, and 34). Related research also confirmed that CetA interacts with CetB (21), indicating that CetB may also play a role in l-fucose chemotaxis. Thus, *C. jejuni*∆*cetB* was constructed, and the chemotaxis assay was performed. The results showed that this mutant is still capable of l-fucose chemotaxis (Fig. 4A). It is known that CetC is a PAS-domain containing homolog of CetB with 63% amino acid identity (30). Also, protein structure predictions of CetB and CetC using AlphaFold2 show similar protein structures. We, therefore, hypothesized that both CetB and CetC may interact with CetA based on the predicted CetC protein structure (Fig. 4B) (33, 34). So, *C. jejuni*∆*cetC* was constructed and it was also capable of l-fucose chemotaxis (Fig. 4A). We next hypothesized that both CetB and CetC play redundant roles in l-fucose chemotaxis, and thus *C. jejuni*∆*cetB*/*cetC* was constructed. The chemotaxis assay showed loss of l-fucose chemotaxis in the double mutant (Fig. 4A), suggesting that swimming in the presence of l-fucose is likely due to energy taxis as transduced through CetB/C and CetA.

### CetAB and CetAC complement the function of the aerotaxis receptor in *E. coli*

We expressed CetA/B and CetA/C in the *E. coli* aerotaxis receptor mutant BT3312 (*E. coli* RP437 ∆*aer-1* ∆*tsr-7021*) and the receptorless mutant BT3388 (*E. coli* RP437 ∆*aer::erm* ∆*tsr-7021* ∆*tar-tap-5201 trg*::Tn*10*) to test if they could complement the function of the aerotaxis receptors. Aer and Tsr are the *E. coli* aerotaxis receptors responsible for the aerotactic response (35–37). Aer contains PAS domains which bind FAD to sense the redox status in the cell (18) and in *C. jejuni*, CetB and CetC are homologs of the *E. coli* Aer PAS domain (19, 21).

Aerotaxis assays with BT3312 and BT3388 complemented with either CetA/B or CetA/C showed significantly increased halo production compared to the mutants (Fig. 4C). This indicates that both CetA/B and CetA/C are able to complement the function of the aerotaxis receptors in *E. coli*. In the BT3312 complements, the halo sizes of CetA/B and CetA/C are similar, while in BT3388, the halo size of CetA/C is significantly smaller than CetA/B, suggesting CetA/B has a dominant role in energy taxis, while CetC may have additional functions in the cell.

### NADPH can be oxidized in the presence of FAD in *C. jejuni*

**Fig 5:**
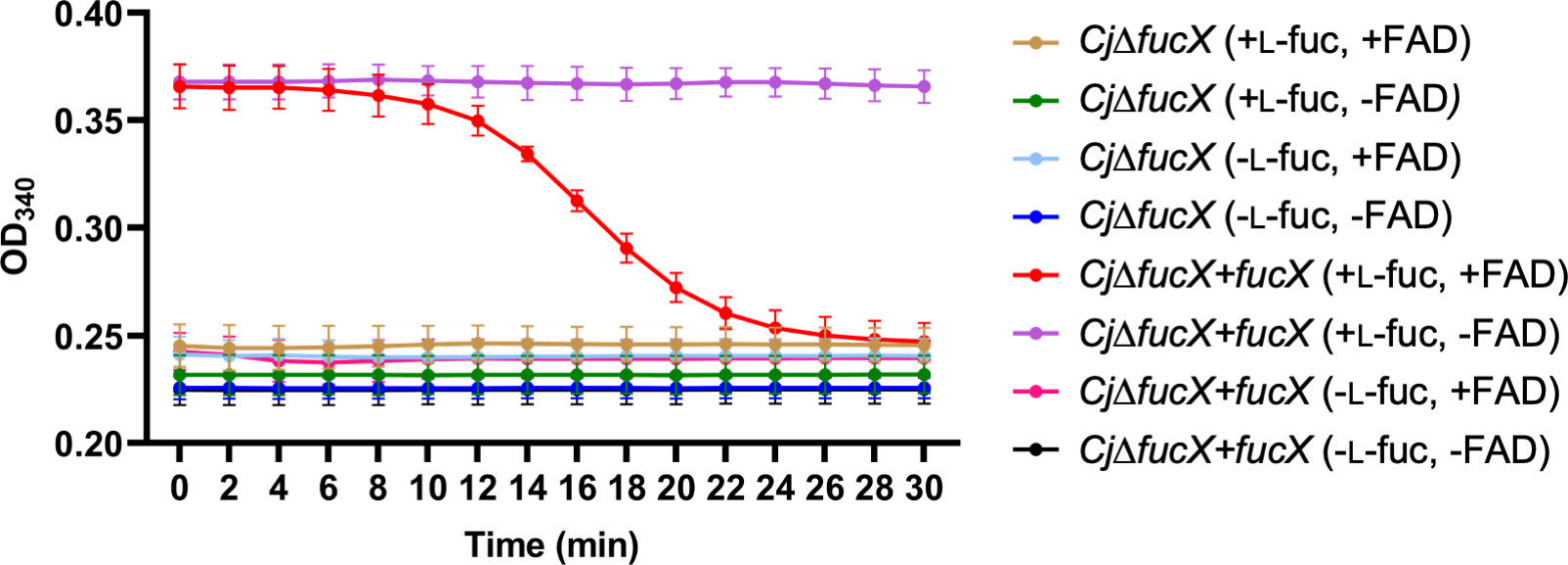
Oxidation of NADPH in the presence of FAD under the indicated conditions. Data points are an average of three biological replicates. OD_340_, absorbance of NADPH.

We hypothesized that NADPH may participate in energy taxis in *C. jejuni,* and FAD is the CetB and CetC cofactor since FAD is the PAS domain cofactor in the *E. coli* Aer system. To test if NADPH can be oxidized in the presence of FAD in *C. jejuni*, we mixed l-fucose, FAD, and NADP^+^ with *C. jejuni*∆*fucX* and *C. jejuni*∆*fucX + fucX* and measured the absorbance of NADPH at OD_340_ (Fig. 5). l-Fucose and NADP^+^ were mixed with the cell lysate first to generate NADPH, as shown in Fig. 5. *C. jejuni*∆*fucX* mixtures did not generate NADPH due to the absence of FucX, while *C. jejuni*∆*fucX + fucX* mixtures show high levels of NADPH when l-fucose is added. Then, FAD was added to some of the samples. *C. jejuni*∆*fucX + fucX* (+L-fuc, +FAD) showed decreased OD_340_, which suggested that NADPH was oxidized in the presence of FAD, while *C. jejuni*∆*fucX + fucX* (+L-fuc, -FAD) still showed high levels of NADPH. The results indicate that *C. jejuni* can reduce FAD in the presence of NADPH, suggesting that NADPH may participate in electron transfer to FAD in the CetABC system.

### *Burkholderia multivorans* FabG restores *C. jejuni* FucX function and plays a role in l-fucose energy taxis

**Fig 6:**
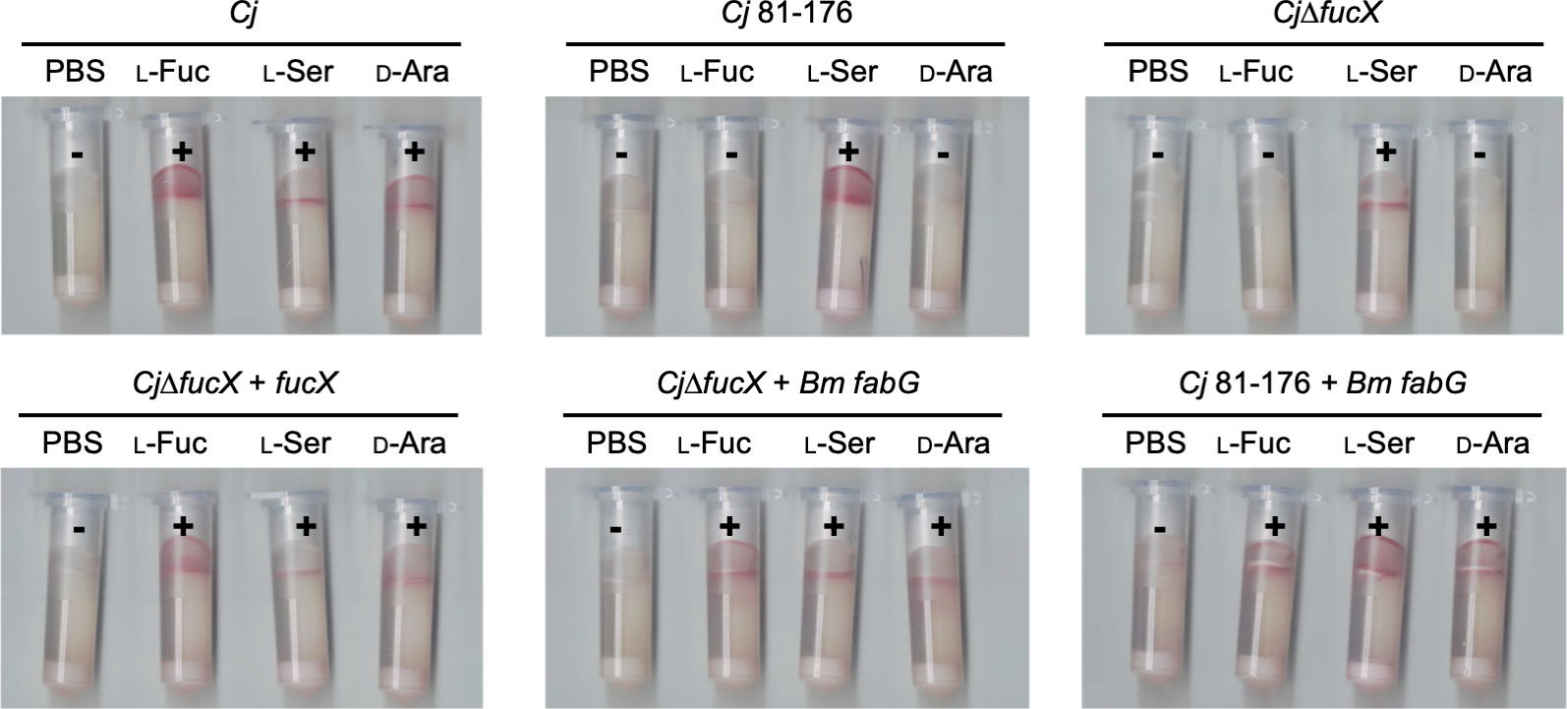
Representative tube-based chemotaxis assay of indicated *C. jejuni* strains. *C. jejuni* 11168 (*Cj*); *C. jejuni* 81–176 (*Cj* 81–176); *C. jejuni* 11168∆*fucX* (*Cj*∆*fucX*); the *Cj* 11168 mutant complemented with *fucX* (+ *fucX*) or the *fabG* homolog from *B. multivorans (+ Bm fabG);* and the *Cj* 81–176 strain complemented with *Bm fabG*. (+), red, positive results; (−), no color, negative results. PBS, negative control; l-Fuc, l-fucose, test compound; l-Ser, l-serine, positive control; d-Ara, d-arabinose, test compound. All assays were repeated at least three times.

Our previous research indicated that the *B. multivorans* FabG is a homolog of *C. jejuni* FucX (8). *B. multivorans* FabG is another l-fucose dehydrogenase capable of oxidizing l-fucose, d-arabinose, and l-galactose (22). To test if *B. multivorans* FabG, like *C. jejuni* FucX, also plays a role in chemotaxis, we introduced *B. multivorans fabG* into *C. jejuni*∆*fucX* and into the non-l-fucose-metabolizing *C. jejuni* strain 81–176. In order to achieve this, it was necessary to codon optimize the sequence of *B. multivorans fabG* for expression in *C. jejuni* since the genome of *B. multivorans* is GC rich (67%), while the genome of *C. jejuni* is AT rich (only 30.5% GC). The sequence of codon-optimized *B. multivorans fabG* is shown in Fig. S3. The results indicate that *B. multivorans fabG* conferred the ability of both *C. jejuni*∆*fucX* and *C. jejuni* 81–176 to swim toward l-fucose and d-arabinose (Fig. 6). The results suggest that other sugar dehydrogenases may also play roles in energy taxis and that this mechanism is more common than originally expected.

## DISCUSSION

Chemotaxis is important in bacterial pathogenesis, and several WHO priority pathogens have chemotaxis systems which assist them in colonization and infection of human hosts (38). To test whether NADPH/NADP^+^ levels influence l-fucose chemotaxis in *C. jejuni*, FucX NADP^+^- and l-fucose-binding site variants were constructed. Our results indicate that the NADP^+^-binding site variant does not swim toward l-fucose, and protein thermal shift assays indicate that this protein (FucX^I19K^) was the least stable based on the low *T_m_* values observed upon NADP^+^ addition in comparison to WT (Table 1; Fig. S1). Chemotaxis results for the l-fucose-binding site variant showed weak l-fucose chemotaxis, and the thermal shift assays confirmed that this mutated protein can bind to NADP^+^ at comparable levels to WT. As expected, dehydrogenase assays using either purified proteins or cell lysates, and growth assays in the presence of l-fucose, confirmed that both binding site mutants could not metabolize l-fucose or show enhanced growth in the presence of this carbon source. These results supported the hypothesis that NADP^+^ binding to FucX could play a role in l-fucose chemotaxis. To further test this hypothesis, we treated the *C. jejuni* catalase mutant, ∆*katA*, with H_2_O_2_ and observed a loss of chemotaxis under these conditions but not when either condition was tested separately. This further supports the importance of microbial sensing of NADPH/NADP^+^ fluctuations in driving l-fucose chemotaxis. However, we cannot exclude the possibilities that additional protein-protein interactions between FucX and other proteins may affect this process, although we did not detect other interactions in our studies.

In the presence of l-fucose, our group previously reported that NADP^+^ (*K*_*M*_: 9.9 ± 1.9 µM) is the preferred FucX cofactor compared to NAD^+^ (*K*_*M*_: 6,050 ± 1,080 µM) (8). We also detected more NADP^+^ than NAD^+^ by NMR in *C. jejuni*, in contrast to the higher levels of NAD^+^ relative to NADP^+^ in *E. coli*. In Epsilonproteobacteria, the roles for NAD^+^ may differ compared to the prototypical *E. coli*. For example, in *H. pylori*, NADPH is the primary electron donor of complex I, while in *C. jejuni*, flavodoxin is the electron donor of complex I (25, 27). It is known that NADP^+^ and NADPH are primarily utilized for anabolic redox reactions, while NAD^+^ and NADH play roles in oxidation reactions (39). These observations suggest that NADP^+^ may possess broader roles in *C. jejuni* than *E. coli,* and the function of NADP^+^ in *C. jejuni* needs further study.

Maschmann et al. reported that the reduction potential of Aer-FAD is −289.6 ± 0.4 mV, and it is high enough to be reduced by various reducing equivalents (including NAD(P)^+^/NAD(P)H, H^+^/H_2_, etc.) generated by NADH dehydrogenase I and II, hydrogenases I and II, among others (40). Our CetA/B and CetA/C aerotaxis complementation results in *E. coli* suggested that the CetB and CetC cofactor may be FAD which is also used in the *E. coli* Aer system. To test if FAD can be reduced in the presence of NADPH in *C. jejuni*, we added FAD to *C. jejuni*∆*fucX + fucX* cell lysates together with l-fucose and NADP^+^, and observed that the amount of NADPH decreased (Fig. 5). The results suggest that NADPH may participate in CetABC energy taxis through reduction of FAD to affect l-fucose chemotaxis. St. Maurice et al. reported that *C. jejuni* FqrB is an NADPH oxidase with strong affinity for quinones, flavodoxin, and NADPH (41). We are currently investigating whether FqrB may participate in this process. We did not detect a difference in NADPH/NADP^+^ ratios between *C. jejuni*∆*fucX* and *C. jejuni*∆*fucX + fucX* (Fig. S1F) and suspect that NADPH was oxidized to NADP^+^ in the presence of cofactors, such as FAD, to maintain cellular homeostasis.

It remains to be determined whether l-fucose chemotaxis directly impacts *C. jejuni* intestinal colonization in breastfed infants. Although *C. jejuni* is a microaerophile which cannot tolerate atmospheric oxygen levels presumably due to its oxygen-labile Fe-S clusters, the bacterium requires oxygen for DNA synthesis (42). It has been shown that successful *C. jejuni* colonization involves swimming along the steep oxygen gradient toward the intestinal crypts, while the anaerobic gut lumen is not only a suboptimal environment for *C. jejuni* growth but also impacts efficient swimming (43–45). Albenberg et al. showed that *Campylobacter* is abundant in mucosal biopsies of both adults and children (aged 3–12) but sparse in stool specimens when comparing microbial populations between these two sites (46). When infants are breastfed, gut commensals such as *Bacteroides* spp. and *Bifidobacterium* spp. are abundant and are capable of secreting an enormous repertoire of glycosidases, including fucosidases, to release l-fucose from HMOs and mucin to support the growth of l-fucose-metabolizing isolates of *C. jejuni* (8, 12). However, our group reported there are significantly higher proportions of non-l-fucose-metabolizing compared to l-fucose-metabolizing *Campylobacters* in the feces of breastfed infants, while the ratios are nearly equal in non-breastfed infants (11). There have also been associations in the literature describing an inverse relationship between l-fucose-metabolizing *C. jejuni* isolates and non-l-fucose-metabolizing *C. jejuni* strains that have an expanded amino acid repertoire capable of also utilizing glutamine and glutathione (47), although this was not observed in our study (8). We now hypothesize that l-fucose chemotaxis plays a role in this process prompting l-fucose-metabolizing *C. jejuni* to swim toward free l-fucose in the intestinal lumen where the low oxygen levels may further impede *C. jejuni* motility and increase clearance of the microbe from the gut (45), while non-l-fucose-metabolizing *C. jejuni* swim toward the intestinal mucosa and cause disease (Fig. S4). We are currently testing this theory in appropriate animal models.

Our study has identified a novel mechanism for *C. jejuni* l-fucose chemotaxis through upregulation of the l-fucose dehydrogenase, FucX, a metabolic enzyme in the fucose/arabinose catabolic pathway. We demonstrate that FucX may alter NADPH/NADP^+^ ratios within the cell leading to the reduction of the predicted FAD cofactor of CetB and CetC and driving l-fucose-metabolizing *C. jejuni* isolates to move toward l-fucose—which would be particularly abundant in the gut lumen of breastfed infants (Fig. S4). Future studies are aimed at examining whether FAD reduction causes conformational changes in the interaction between CetB/CetC and CetA which ultimately trigger a change in flagellar rotation. In addition, this mechanism of chemotaxis is likely utilized by the opportunistic pathogen *B. multivorans*, where FabG (which oxidizes l-fucose, d-arabinose, and l-galactose) may play an important role in lung colonization of cystic fibrosis patients who produce excessive amounts of mucus comprising at least two of these monosaccharides (22). By understanding l-fucose-related chemotaxis in *C. jejuni*, we may be able to better understand how *C. jejuni* colonizes the intestinal tract of breastfed infants and elucidate the mechanism for selection of *C. jejuni* isolates with improved colonization potential. Future work will examine the effect of human breastmilk on *C. jejuni* intestinal colonization in animal models of disease that we are currently developing.

## MATERIALS AND METHODS

### Strains, plasmids, and growth conditions

*C. jejuni* strains were grown on Mueller Hinton (MH) agar plates (Criterion) and in minimum essential medium α (MEMα, Gibco) at 37°C under microaerobic conditions (85% N_2_, 10% CO_2_, 5% O_2_). *E. coli* strains were grown on Luria-Bertani (LB, Criterion) agar plates or broth at 37°C under aerobic conditions. If required, antibiotics were added to a final concentration of 50 µg/mL for kanamycin, 25 µg/mL for chloramphenicol, 100 µg/mL for ampicillin, 10 µg/mL for tetracycline, 25 µg/mL for trimethoprim, and 200 µg/mL for erythromycin. All strains, plasmids, and primers used in this study are listed in Tables S2 to S4. All *C. jejuni* strains were constructed in *C. jejuni* NCTC11168, unless otherwise indicated.

### Construction of *C. jejuni* mutants

Variants of *C. jejuni*∆*fucX* disrupting either NADP^+^- (I19K) or l-fucose- (Q147S) binding sites were constructed using primers CS-597 + CS-598 and CS-940 + CS-939 to mutagenize previously constructed pRRK:*fucX* (7) and pET30a(+)-*fucX* (8) using the QuickChange XL Site-Directed Mutagenesis Kit (Agilent Technologies) according to manufacturer’s instructions and electroporated into *C. jejuni*∆*fucX*. The transformants were verified by PCR and Sanger sequencing.

The plasmids used to construct *C. jejuni tlp* mutants are listed in Table S3. The plasmids were electroporated into *C. jejuni,* and the transformants were identified by PCR.

To express the *Burkholderia multivorans fabG* in *C. jejuni*, CS-814 + CS-761 were used to amplify *Bm fabG* from pUC57-codon-optimized *Bm fabG* (GenScript) for ligation into pRRK plasmid at MfeI and XbaI sites. To express *C. jejuni cetA*/*B* and *cetA*/*C* into *E. coli* BT3312 (∆*aer-1* ∆*tsr-7021*) and BT3388 (∆*aer::erm* ∆*tsr-7021* ∆*tar-tap-5021 trg*::Tn*10*), CS-1081 + CS-1082 and CS-1079 + CS-1080 were used to amplify *Cj cetA*/*B* and *cetA*/*C* for ligation into the pMLBAD plasmid at the EcoRI and PstI sites. The plasmids were verified by PCR and Sanger sequencing, and the transformants were identified by PCR.

### SDS-PAGE and western blotting

The purified proteins were separated on 12.5% separating/5% stacking polyacrylamide gels at 15 mA for 1 hour. One gel was stained with Commassie brilliant blue R250, and the other was transferred to nitrocellulose at 100 V for 1 hour. The membrane was blocked in 2% skim milk in 1× phosphate-buffered saline with Tween-20 (PBST) for 1 hour. Then, the FucX antisera (see below) were added at a dilution of 1:5,000 and incubated overnight at 4°C. The membrane was washed three times with 2% skim milk (in PBST), then goat-anti-rabbit-HRP was added at a dilution of 1:5,000, and incubated for 1 hour. After the membrane was washed with 2% skim milk (in PBST) for three times, the BioRad Clarity Western ECL substrate was used to visualize the proteins according to the manufacturer’s instructions.

FucX antibodies were generated by immunizing two rabbits with approximately 600–700 ng of protein followed by two boosters according to the approved University of Alberta Animal Care Committee Protocol AUP00000964. To generate highly specific FucX-antisera, purified FucX was applied to Ni-NTA columns followed with two washes with 1× phosphate-buffered saline (PBS). Then, 200 µL anti-FucX rabbit serum was diluted in 200 µL PBS and applied to the column followed by three washes with five column volumes of PBS. To elute the antibody from the column, three volumes of 1 mL 0.1 M glycine-HCl (pH 2.5–3.0) were applied to the column with addition of 1/10th of the elution volume of 1M Tris-HCl (pH 8.5) to neutralize the pH. The antibody was dialyzed against PBS at 4°C overnight.

### Aerotaxis assay

The aerotaxis assay was performed as described (48). In brief, minimal semisolid agar medium containing 30 mM sodium succinate and appropriate antibiotics was prepared, and cells were inoculated into the agar. l-Arabinose (0.5% final concentration) was added on the plates for CetA/B and CetA/C complementary *E. coli* strains. The plates were incubated at 30°C under humid conditions for 3 days.

### NADPH/NADP^+^ ratio measurements

*C. jejuni* strains were grown on MH agar plates at 37°C under microaerobic conditions for 2 days, then the cells were restreaked to MH agar plates supplemented with necessary antibiotics, and incubated at 37°C overnight. The next day, cells were harvested in MH broth. The cells were adjusted to OD_600_ = 1, and 1 mL cell suspension was transferred to 5 mL MH broth shaking at 200 rpm at 37°C under microaerobic conditions for 4 hours. The final concentration of H_2_O_2_ (ThermoFisher) was 0.001%. *E. coli* K12 was processed the same as *C. jejuni* but incubated in LB broth under aerobic conditions . After 4 hours, the cell cultures were lysed immediately using the lysis buffer, and the NADPH/NADP^+^ measurement kit (Promega) was used following the manufacturer’s instructions.

### Tube-based chemotaxis assay

A detailed method for this assay is described elsewhere (7). In brief, cells were harvested and washed three times with PBS, then resuspended in 0.4% PBS agar (1 g cell pellet suspended in 2.8 mL agar). Then, 250 µL of cell suspension was allowed to solidify at the bottom of a 2-mL centrifuge tube with 1 mL PBS agar added on top. In the *C. jejuni*∆*katA* tube-based chemotaxis assay, H_2_O_2_ was mixed with the top 1 mL PBS agar to a final concentration of 0.001%. Paper disks soaked with 50 µL of the 1M test compounds were added to the top of the tubes. The tubes were incubated at 37°C for 3 days. Subsequently, 200 µL of 0.01% 2,3,5-triphenyltetrazolium chloride (GoldBio) was added on top of the tubes and incubated at 37°C under microaerobic conditions for 2 hours. The formation of a red ring indicates a positive result.

### Dehydrogenase assay

*C. jejuni* whole-cell lysates were generated by mixing B-PER chemical lysis solution (ThermoFisher) and proteinase inhibitor (Roche) with the cell pellet following the manufacturer’s instructions and centrifuged at 22,000 × *g* (VWR, 2405–37) for 10 minutes at 4°C to remove debris. The protein concentrations in the supernatant were measured using the BCA kit (ThermoFisher). The dehydrogenase assay reaction mixture was set up as described (8). In brief, 5 µg of cell lysate, 1 mM MgCl_2_, 100 µM NADP^+^ (Enzo Life Sciences), and 1 mM l-fucose (Biosynth) were mixed in 50 mM Tris-HCl (pH 7.5) to final volume of 100 μL, and OD_340_ was measured for 20 minutes in kinetics mode in a BioTek plate reader at RT.

Overexpressed His-tagged FucX and the variants were purified from *E. coli* BL21 using Nickel-NTA agarose beads (GoldBio) as previously described (8), with one modification to desalt in imidazole-free buffer at 4°C overnight rather than using PD-10 columns. Then, 5 nM of protein was used for the dehydrogenase assay as described for the cell lysates.

### Thermal shift assay

Purified FucX proteins were diluted to final concentration to 20 µM. To set up the reaction mixture, 10 µM protein, 100 µM NADP^+^ (substituting H_2_O in no ligand groups), 1 mM MgCl_2_, and 5× SYPRO Orange Dye (Invitrogen) were mixed together to a final volume of 20 µL and added to Hard-Shell 96-Well PCR Plates (Bio-Rad), with three technical replicates per biological replicate. Plates were placed in the CFX96 Touch Real-Time PCR System (Bio-Rad) for a standard protein melt curve experiment, with a temperature scan from 20 to 90°C at 1°C per minute, and the results were analyzed using CFX Maestro Software (Bio-Rad).

### l-Fucose growth assay

The minimal l-fucose growth assay was described previously (8). In brief, *C. jejuni* was grown in MEMα supplemented with 20 µM FeSO_4_, with or without 25 mM l-fucose at a starting OD_600_ = 0.05. OD_600_ was measured after 18 hours incubation at 150 rpm, 37°C under microaerobic conditions.

### NMR measurements

To prepare the cell lysates for NMR measurement, 1 mL of ice-cold water and methanol (1:5, vol/vol) was added immediately on MH (for *C. jejuni*) and LB (for *E. coli* K12) agar plates to harvest and suspend the overnight cell cultures, and the samples were incubated on dry ice for 30 minutes before being centrifuged at 22,000 × *g* for 15 minutes at 4°C. The supernatants were dried using nitrogen airflow, and the samples were stored in −80°C until measurement (49).

β-NAD^+^ (Sigma-Aldrich), NADP^+^, and NADPH (Enzo Life Sciences) pure compounds (1–4 mg) were each dissolved in 510 µL D_2_O (99.9% D, Sigma). Then, 10 nmol DSS-d_6_ (Cambridge Isotope Laboratories) was added, and the solution was transferred into a 5-mm NMR tube. The dry material of each extract was dissolved in 510 µL D_2_O (99.9% D), 30 nmol DSS-d_6_ was added, and the solution was transferred into a 5-mm NMR tube. The pD of NAD^+^, NADP^+^, NADPH standards , *C. jejuni* 11168, and *C. jejuni*Δ*fucX* extract samples was adjusted to 7.0 with NaOD or DCl in order to match the pD of the *E. coli* K12 extract sample. The pD was measured using a combined micro-electrode (accumet, Fisherbrand). NMR data were acquired at 25°C on a Varian VNMRS (^1^H, 599.66 MHz) or Bruker Avance III (^1^H, 600.13 MHz) spectrometer each equipped with a cold probe. Semi-quantitative ^1^H data were acquired with spectral width of 10,823 Hz, 16,384 complex data points, 60° pulse flip angle, and total recycle delay of 3.7 s between each scan. The number of scans was 8 for the NAD^+^, NADP^+^, and NADPH standards, 1,880 for the *E. coli* K12 extract, 15,280 for *C. jejuni* Δ*fucX*, and 20,480 for *C. jejuni* 11168. The 2D TOCSY data were recorded with spectral widths of 5,388 Hz in both dimensions, 128 × 2,048 (*f*1 × *f*2) complex data vectors, and 4 (NADP^+^ and NADPH) or 64 (*E. coli* K12) scans per increment. ^1^H chemical shifts were referenced to the signal of DSS-d_6_ at 0.00 ppm. The spectra were processed and analyzed with MestreNova (version 14.1.1). Mean integrals of resolved NAD^+^ (9.32, 9.13 ppm) and NADP^+^ (9.28, 9.09 ppm) signals were used to determine approximate molar ratios of the two coenzymes.

### FAD reduction assay

*C. jejuni* was harvested in PBS and sonicated, then cell lysates were centrifuged at 22,000 × *g* for 15 minutes at 4°C to remove debris. The protein concentrations were measured using the BCA Assay Kit, and concentrations were adjusted to 1 mg/mL. Cell lysates (20 µg) were mixed with 100 µM NADP^+^, 1 mM MgCl_2_, 50 mM Tris buffer (pH 7.5), and 1 mM l-fucose (H_2_O for non-l-fucose groups) and incubated at RT for 10 minutes to generate NADPH. Before measurement, 20 µM FAD (Enzo Life Sciences) was added to FAD groups (H_2_O for non-FAD groups), followed by measurements at OD_340_ in kinetics mode in the BioTek plate reader for 30 minutes at RT.

## SUPPLEMENTAL MATERIAL

Supplemental Material mbio.02732-23-s0001.pdf Supplemental figures and tables.

ASM does not own the copyrights to Supplemental Material that may be linked to, or accessed through, an article. The authors have granted ASM a non-exclusive, world-wide license to publish the Supplemental Material files. Please contact the corresponding author directly for reuse.
