# Supplementary material for: Campylobacter jejuni uses energy taxis and a dehydrogenase enzyme for l-fucose chemotaxis
Source: mBio. 2023 Nov 30;14(6):e02732-23. doi: 10.1128/mbio.02732-23 (PMC10746189; doi:10.1128/mbio.02732-23)
Supplement: Supplemental Material — Supplemental figures and tables. [file mbio.02732-23-s0001.pdf]

Campylobacter jejuni uses energy taxis and a dehydrogenase enzyme for L-fucose chemotaxis

Bibi Zhou[a,b], Jolene M. Garber[a,b*], Jiri Vlach[b], Parastoo Azadi[b], Kenneth K. S. Ng[c], Jorge C. Escalante-Semerena[a], Christine M. Szymanski[a,b#]

[a]Department of Microbiology, University of Georgia, Athens, GA, USA.

[b]Complex Carbohydrate Research Center, University of Georgia, Athens, GA, USA.

[c]Department of Chemistry and Biochemistry, University of Windsor, Windsor, ON, Canada

[*]Current address:

Department of Medical Microbiology and Immunology, University of Alberta, Edmonton, Alberta T6G2E9, Canada

This file includes:

Figure S1. Further characterization of FucX variants and NADPH/NADP$^+$ ratios.

Figure S2. Tube-based chemotaxis assays of *C. jejuni tlp* mutants.

Table S1. Molar ratios of NADP$^+$ and NAD$^+$ in *E. coli* K12 and *C. jejuni* cell extracts based on ^{1}H NMR results.

Table S2. Strains used in this study.

Table S3. Plasmids used in this study.

Table S4. Primers used in this study.

Figure S3. *Burkholderia multivorans* FabG.

Figure S4. Comparison of the *E. coli* Aer system with the *C. jejuni* CetABC components demonstrating how colonization could be impacted by L-fucose sensing.

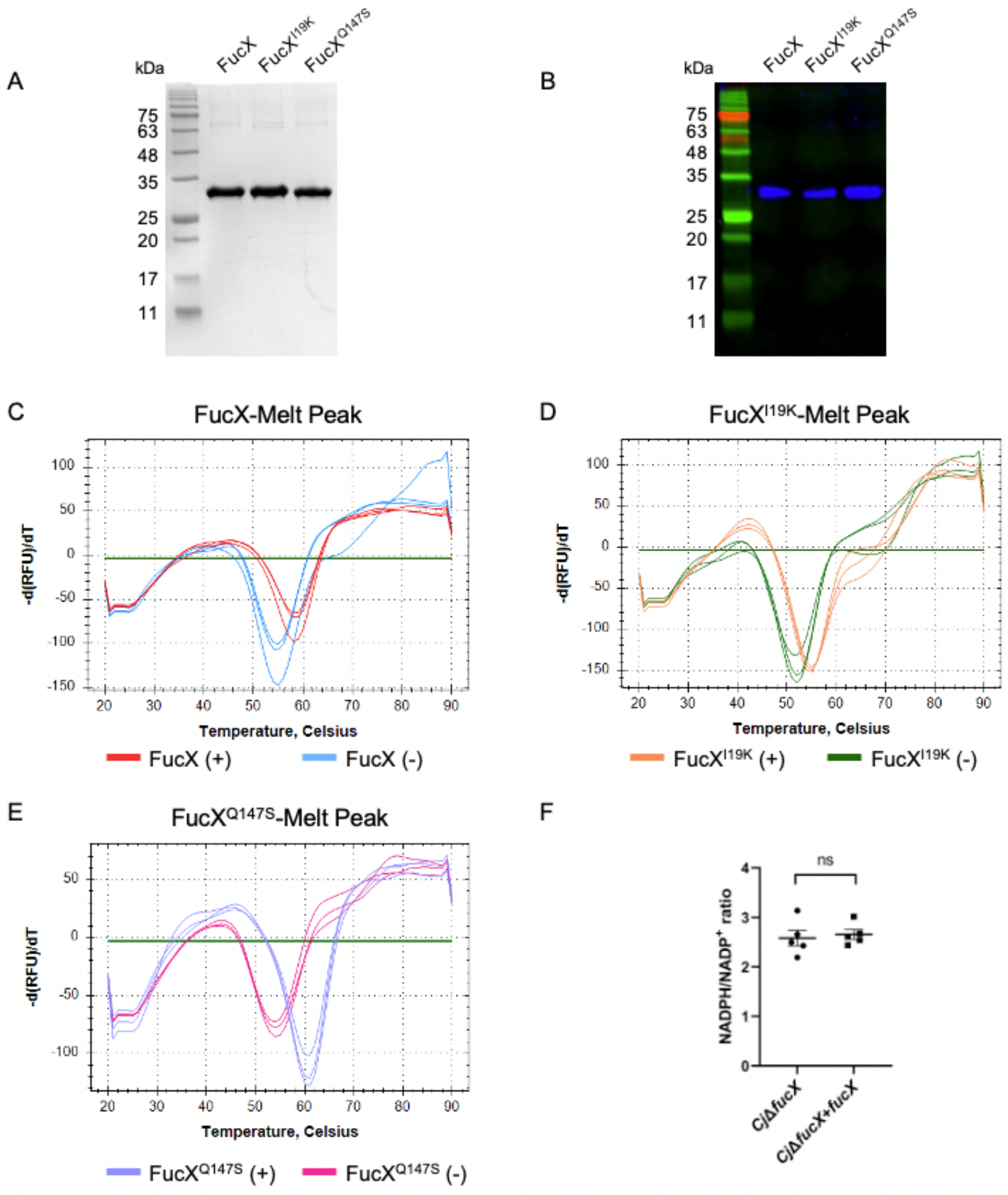

FIG S1 Further characterization of FucX variants and NADPH/NADP$^+$ ratios. (A) Coomassie-blue stained SDS-PAGE of purified FucX, FucX^{I19K} and FucX^{Q147S}. (B) Purified FucX, FucX^{I19K} and FucX^{Q147S} detected by western blotting using anti-FucX

antisera. (C, D, E) Representative thermal shift assay melting curves for FucX, FucX^{I19K}, FucX^{Q147S} with and without the NADP$^+$ ligand. (+): with NADP$^+$; (-) without NADP$^+$. FucX: WT; FucX^{I19K}: NADP$^+$ binding site mutant; FucX^{Q147S}: L-fucose binding site mutant. All assays were repeated at least three times. (F) NADPH/NADP$^+$ ratio of *C. jejuni*Δ*fucX* and *C. jejuni*Δ*fucX*+*fucX* using the Promega NADPH/NADP$^+$ measurement kit, with each data point representing one biological replicate. Error bars represent the standard error of the mean. ns, not significant, determined by the Student's t test.

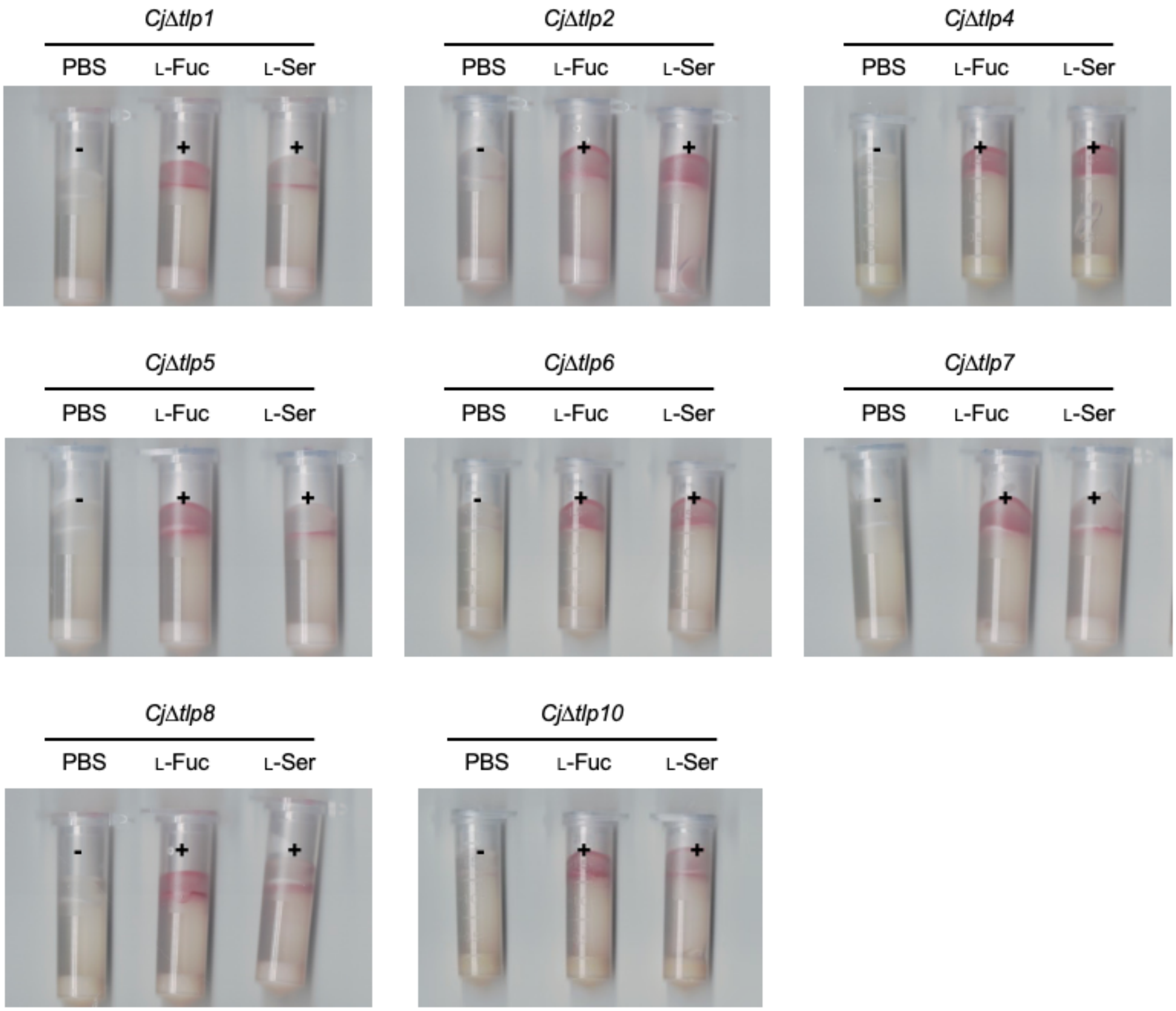

FIG S2 Tube-based chemotaxis assays of *C. jejuni tlp* mutants. (+): red, positive result; (-): no color, negative result. PBS: negative control; L-Fuc: L-fucose, test compound; L-Ser: L-serine, positive control. All assays were repeated at least three times.

Table S1 Molar ratios of $NADP^+$ and NAD^+ in *E. coli* K12, *C. jejuni* 11168 and *C. jejuni* 11168Δ*fucX* cell extracts based on ^1H NMR results.

Compound	Molar ratio		
	E. coli K12	*C. jejuni*	*C. jejuni*Δ*fucX*
$NADP^+$	20.7	89	79.5
NAD^+	79.3	11[†]	20.5

[†]Molar ratio for *C. jejuni* has larger uncertainty due to low signal-to-noise ratio of the NAD^+ signals.

Table S2 Strains used in this study.

**Strains**	**Description/genotype**	**Source**
C. jejuni NCTC11168	Clinical isolate used for genome sequencing	(1)
C. jejuni 81-176	Clinical isolate	(2)
C. jejuni NCTC11168Δ*fucX*	*C. jejuni* NCTC11168 *fucX* mutant, Cm^{R}	(3)
C. jejuni NCTC11168Δ*fucX*+*fucX*	*C. jejuni* NCTC11168 *fucX* complementary strain, Kan^{R}, Cm^{R}.	(3)
C. jejuni NCTC11168Δ*fucX*+*fucX*^{I19K}	*C. jejuni* NCTC11168 FucX NADP^{+} binding site mutant, Kan^{R}, Cm^{R}.	This study
C. jejuni NCTC11168Δ*fucX*+*fucX*^{Q147S}	*C. jejuni* NCTC11168 FucX L-fucose binding site mutant, Kan^{R}, Cm^{R}.	This study
C. jejuni 81-176+*fucX*	Expressing FucX in non-L-fucose utilizing *C. jejuni*, Kan^{R}.	(4)
C. jejuni NCTC11168Δ*katA*	*C. jejuni* NCTC11168 catalase mutant, Cm^{R}.	(5)
C. jejuni NCTC11168Δ*tlp1* (*cj1506c*)	*C. jejuni* NCTC11168 *tlp1* mutant, Cm^{R}.	This study
C. jejuni NCTC11168Δ*tlp2* (*cj0144*)	*C. jejuni* NCTC11168 *tlp2* mutant, Cm^{R}.	This study
C. jejuni NCTC11168Δ*tlp4* (*cj0262c*)	*C. jejuni* NCTC11168 *tlp4* mutant, Cm^{R}.	This study
C. jejuni NCTC11168Δ*tlp5* (*cj0246c*)	*C. jejuni* NCTC11168 *tlp5* mutant, Kan^{R}.	This study
C. jejuni NCTC11168Δ*tlp6* (*cj0448c*)	*C. jejuni* NCTC11168 *tlp6* mutant, Kan^{R}.	This study
C. jejuni NCTC11168Δ*tlp7* (*cj0951c*)	*C. jejuni* NCTC11168 *tlp7* mutant, Kan^{R}.	This study
C. jejuni NCTC11168Δ*tlp8* (*cj1110c*, *cetZ*)	*C. jejuni* NCTC11168 *tlp8* mutant, Cm^{R}.	This study
C. jejuni NCTC11168Δ*cetA* (*cj1190c*, *tlp9*)	*C. jejuni* NCTC11168 *cetA* mutant, Cm^{R}.	This study
C. jejuni NCTC11168Δ*tlp10* (*cj0019c*)	*C. jejuni* NCTC11168 *tlp10* mutant, Cm^{R}.	This study
C. jejuni NCTC11168Δ*cetB* (*cj1189*)	*C. jejuni* NCTC11168 *cetB* mutant, Cm^{R}.	This study
C. jejuni NCTC11168Δ*cetC* (*cj1191c*)	*C. jejuni* NCTC11168 *cetC* mutant, Cm^{R}.	This study
C. jejuni NCTC11168Δ*cetB*/cetC	*C. jejuni* NCTC11168 *cetB/C* double mutant, Kan^{R}, Cm^{R}.	This study
E. coli Top10	F- *mcrA (mrr-hsdRMS-mcrBC) 80lacZ M15 lacX74 recA1 ara 139 (ara-leu)7697 galU galK rpsL (StrR) endA1 nupG*	New England BioLabs
E. coli BL21 (DE3)	*fhuA2 [lon] ompT gal (λ DE3) [dcm] ΔhsdS λ DE3 = λ sBamHIo ΔEcoRI-B int::(lacI::PlacUV5::T7 gene1) i21 Δnin5*	New England BioLabs
E. coli K12	F- lambda- *ilvG*- *rfb*-50 *rph*-1	(6)
E. coli RP437	F-, *thr-1, araC14, leuB6(Am), fhuA31, lacY1, tsx-78, λ-, eda-50, hisG4(Oc), rfbC1, rpsL136(strR), xylA5, mtl-1, metF159(Am), thiE1*	(7)
E. coli BT3312	*E. coli* RP437 *Δaer-1 Δtsr-7021*	(8)
E. coli BT3388	*E. coli* RP437 Δ*aer*::*erm* Δ*tsr-7021* Δ*tar-tap-5201 trg*::Tn*10*, Tet^{R}, Ery^{R}.	(9)
E. coli BT3312+pGH1 (Aer)	*E. coli* BT3312 complemented with WT Aer, Amp^{R}.	(10)
E. coli BT3388+pGH1 (Aer)	*E. coli* BT3388 complemented with WT Aer, Amp^{R}.	(10)
E. coli BT3312+*cetA/B*	*E. coli* BT3312 complemented with *C. jejuni cetA/B*, Tet^{R}, Ery^{R}, Tmp^{R}.	This study
E. coli BT3388+*cetA/B*	*E. coli* BT3388 complemented with *C. jejuni cetA/B*, Tmp^{R}.	This study

E. coli BT3312+*cetA/C*	*E. coli* BT3312 complemented with *C. jejuni cetA/C*, Tet^R, Ery^R, Tmp^R.	This study
E. coli BT3388+*cetA/C*	*E. coli* BT3388 complemented with *C. jejuni cetA/C*, Tmp^R.	This study

Table S3 Plasmids used in this study.

**Plasmid**		
pRRK	*E. coli* cloning vector used for complementation of *C. jejuni* strains, Amp^R Kan^R.	(11)
pRRK: *fucX*	Use as the vector for Q147S, I19K plasmid construction, Amp^R Kan^R.	(3)
pUC57-codon-optimized *B.multivorans fabG*	Plasmid from Genscript with codon-optimized *Bm fabG*, Amp^R.	This study
pRRK: *Bm fabG*	To express *Bm fabG* into *C. jejuni* 11168Δ*fucX* and *C. jejuni* 81-176, Amp^R Kan^R.	This study
pRRK: *fucX* Q147S	To construct *fucX* L-fucose binding site mutant, Amp^R Kan^R.	This study
pRRK: *fucX* I19K	To construct *fucX* $NADP^+$ binding site mutant, Amp^R Kan^R.	This study
pET30a(+)-*fucX*	To express FucX protein.	(4)
pET30a(+)-*fucX* I19K	To express FucX I19K protein.	This study
pET30a(+)-*fucX* Q147S	To express FucX Q147S protein.	This study
pMLBAD	Vector optimized for inducible gene expression in *Burkholderia*, Tmp^R.	(12)
pMLBAD-cetC/A	To express *C. jejuni cetC* and *cetA* in *E. coli*, Tmp^R.	This study
pMLBAD-cetA/B	To express *C. jejuni cetA* and *cetB* in *E. coli*, Tmp^R.	This study
pDRH811	To knock out *tlp1* in *C. jejuni* NCTC11168, Cm^R.	(13)
pDRH1001	To knock out *tlp2* in *C. jejuni* NCTC11168, Cm^R.	From David Hendrixson
pDRH808	To knock out *tlp4* in *C. jejuni* NCTC11168, Cm^R.	(13)
pBZ040	To knock out *tlp5* in *C. jejuni* NCTC11168, Kan^R.	This study
pDRH838	To knock out *tlp6* in *C. jejuni* NCTC11168, Kan^R.	(13)
pBZ043	To knock out *tlp7* in *C. jejuni* NCTC11168, Kan^R.	This study
pDRH810	To knock out *tlp8* in *C. jejuni* NCTC11168, Cm^R.	(13)
pDRH301	To knock out *cetA* in *C. jejuni* NCTC11168, Cm^R.	From David Hendrixson
pDRH1006	To knock out *tlp10* in *C. jejuni* NCTC11168, Cm^R.	(13)
pDRH278	To knock out *cetB* in *C. jejuni* NCTC11168, Cm^R.	From David Hendrixson
pDRH305	To knock out *cetC* in *C. jejuni* NCTC11168, Cm^R.	(13)
pBZ058	To knock out whole *cetC* in *C. jejuni* NCTC11168, Kan^R.	This study

Table S4 Primers used in this study.

**Primers**	Oligosaccharides	Sources
fucX I19K-F (CS-597)	ATAATTTAGCAATACCATACCCCTTACCTTTAGCCCCTCCTGTAATAATAC	This study
fucX I19K-R (CS-598)	GTATTATTACAGGAGGGGCTAAAGGTAAGGGGTATGGTATTGCTAAATTAT	This study
fucX Q147S-F (CS-940)	ACAGGCATCACAGGATCAGGAAGAACTTCAGCTTATGCTTC	This study
fucX Q147S-R (CS-939)	AGCTGAAGTTCTTCCTGATCCTGTGATGCCTGTTTTACTC	This study
XbaI-*B. multivorans fabG*-F (CS-814)	GCATCTAGAAAGAAGGAGATATACATGGATCTTAATTTACAAGATAAAG	This study
MfeI-*B. multivorans fabG*-R (CS-761)	GCACAATTGTTAGTGGTGATGGTGATGATGAACTAAAGCTCTATCTAAATG	This study
EcoRI-*cetC*-F (CS-1079)	GCAGAATTCACCATGAAAGAAATAGTTTTGTC	This study
PstI-*cetA*-R (CS-1080)	GCACTGCAGTTAGTGGTGATGGTGATGATGTATTTTTAATTTTGCTAAG	This study
EcoRI-*cetA*-F (CS-1081)	GCAGAATTCACCATGGTGAAAAATGGATTATTG	This study
PstI-*cetB*-R (CS-1082)	GCACTGCAGTTAGTGGTGATGGTGATGATGTTTAGCTTCTTGAAGAG	This study

1	2	3	4	5	6	7	8	9	10	11	12	13	14	15	16
M	D	L	N	L	Q	D	K	V	V	I	V	T	G	G	A
GTG	GAT	CTG	AAT	CTG	CAG	GAC	AAG	GTC	GTG	ATC	GTG	ACG	GGC	GGC	GCG
ATG	GAT	CTT	AAT	TTA	CAA	GAT	AAA	GTT	GTT	ATT	GTT	ACT	GGA	GGA	GCT

17	18	19	20	21	22	23	24	25	26	27	28	29	30	31	32
S	G	I	G	G	A	I	S	M	R	L	A	E	E	R	A
TCG	GGC	ATC	GGC	GGC	GCG	ATC	TCG	ATG	CGG	CTC	GCG	GAA	GAA	CGC	GCG
AGT	GGA	ATT	GGA	GGA	GCT	ATT	AGT	ATG	AGA	TTA	GCT	GAA	GAA	AGA	GCT

33	34	35	36	37	38	39	40	41	42	43	44	45	46	47	48
I	P	V	V	F	A	R	H	A	P	D	G	A	F	L	D
ATT	CCG	GTC	GTG	TTC	GCG	CGC	CAC	GCG	CCC	GAC	GGC	GCG	TTC	CTC	GAC
ATT	CCT	GTT	GTT	TTT	GCT	AGA	CAT	GCT	CCT	GAT	GGT	GCA	TTT	TTA	GAT

49	50	51	52	53	54	55	56	57	58	59	60	61	62	63	64
A	L	A	Q	R	Q	P	R	A	T	Y	L	P	V	E	L
GCG	CTC	GCG	CAA	CGC	CAG	CCG	CGC	GCG	ACG	TAT	CTG	CCG	GTC	GAA	CTG
GCT	TTA	GCT	CAA	AGA	CAA	CCT	AGA	GCT	ACT	TAT	TTA	CCT	GTT	GAA	CTT

65	66	67	68	69	70	71	72	73	74	75	76	77	78	79	80
Q	D	D	A	Q	C	R	D	A	V	A	Q	T	I	A	T
CAG	GAC	GAT	GCG	CAA	TGC	CGC	GAC	GCG	GTC	GCG	CAG	ACG	ATC	GCG	ACG
CAA	GAT	GAT	GCT	CAA	TGT	AGA	GAT	GCT	GTT	GCT	CAA	ACT	ATT	GCT	ACT

81	82	83	84	85	86	87	88	89	90	91	92	93	94	95	96
F	G	R	L	D	G	L	V	N	N	A	G	V	N	D	G
TTC	GGC	CGC	CTC	GAC	GGG	CTC	GTG	AAC	AAC	GCA	GGC	GTC	AAC	GAC	GGC
TTT	GGA	AGA	TTA	GAT	GGT	CTT	GTT	AAT	AAT	GCT	GGA	GTT	AAT	GAT	GGA

97	98	99	100	101	102	103	104	105	106	107	108	109	110	111	112
I	G	L	D	A	G	R	D	A	F	V	A	S	L	E	R
ATC	GGC	CTC	GAC	GCG	GGG	CGC	GAC	GCG	TTC	GTC	GCA	TCG	CTC	GAG	CGC
ATT	GGT	TTA	GAT	GCT	GGT	AGA	GAT	GCT	TTT	GTT	GCT	AGT	TTA	GAA	AGA

113	114	115	116	117	118	119	120	121	122	123	124	125	126	127	128
N	L	I	H	Y	Y	A	M	A	H	Y	C	V	P	H	L
AAC	CTG	ATC	CAC	TAC	TAC	GCG	ATG	GCG	CAT	TAC	TGC	GTG	CCG	CAT	CTG
AAT	CTT	ATT	CAT	TAT	TAT	GCT	ATG	GCT	CAT	TAT	TGT	GTT	CCT	CAT	CTT

129	130	131	132	133	134	135	136	137	138	139	140	141	142	143	144
K	A	T	R	G	A	I	V	N	I	S	S	K	T	A	V
AAG	GCG	ACG	CGC	GGC	GCG	ATC	GTC	AAC	ATT	TCG	TCG	AAG	ACG	GCC	GTG
AAA	GCT	ACT	AGA	GGA	GCT	ATT	GTT	AAT	ATT	AGT	AGT	AAA	ACT	GCT	GTT

145	146	147	148	149	150	151	152	153	154	155	156	157	158	159	160
T	G	Q	G	N	T	S	G	Y	C	A	S	K	G	A	Q
ACC	GGG	CAG	GGC	AAC	ACG	AGC	GGC	TAT	TGC	GCA	TCG	AAG	GGC	GCG	CAG
ACT	GGA	CAA	GGT	AAT	ACT	AGT	GGA	TAT	TGT	GCT	AGT	AAA	GGT	GCT	CAA

161	162	163	164	165	166	167	168	169	170	171	172	173	174	175	176
L	A	L	T	R	E	W	A	V	A	L	R	E	H	G	V
CTC	GCG	CTG	ACG	CGC	GAA	TGG	GCG	GTC	GCG	CTG	CGC	GAA	CAC	GGC	GTG
TTA	GCT	TTA	ACT	AGA	GAA	TGG	GCT	GTT	GCT	TTA	AGA	GAA	CAT	GGT	GTT

177	178	179	180	181	182	183	184	185	186	187	188	189	190	191	192
R	V	N	A	V	I	P	A	E	V	M	T	P	L	Y	R
CGC	GTG	AAC	GCG	GTG	ATT	CCG	GCC	GAG	GTC	ATG	ACG	CCG	CTC	TAC	CGG
AGA	GTT	AAT	GCT	GTT	ATT	CCT	GCT	GAA	GTT	ATG	ACT	CCT	TTA	TAT	AGA

193	194	195	196	197	198	199	200	201	202	203	204	205	206	207	208
N	W	I	A	T	F	E	D	P	E	A	K	L	A	E	I
AAC	TGG	ATC	GCG	ACG	TTC	GAG	GAT	CCC	GAG	GCG	AAG	CTC	GCC	GAG	ATC
AAT	TGG	ATT	GCT	ACT	TTT	GAA	GAT	CCT	GAA	GCT	AAA	CTT	GCT	GAA	ATT

209	210	211	212	213	214	215	216	217	218	219	220	221	222	223	224
A	A	K	V	P	L	G	R	R	F	T	T	P	D	E	I
GCG	GCG	AAG	GTG	CCG	CTC	GGC	AGG	CGC	TTC	ACG	ACG	CCG	GAC	GAG	ATC
GCT	GCT	AAA	GTT	CCT	TTA	GGA	AGA	AGA	TTT	ACT	ACT	CCT	GAT	GAA	ATT

225	226	227	228	229	230	231	232	233	234	235	236	237	238	239	240
A	D	T	A	V	F	L	L	S	P	R	A	S	H	T	T
GCC	GAT	ACG	GCC	GTG	TTC	CTG	CTG	TCG	CCG	CGC	GCG	TCG	CAC	ACG	ACG
GCT	GAT	ACT	GCT	GTA	TTT	TTA	CTT	AGT	CCT	AGA	GCT	AGT	CAT	ACT	ACT

241	242	243	244	245	246	247	248	249	250	251	252	253	254	255	256
G	E	W	L	F	V	D	G	G	Y	T	H	L	D	R	A
GGC	GAA	TGG	CTG	TTC	GTC	GAC	GGC	GGC	TAT	ACG	CAT	CTC	GAT	CGT	GCG
GGT	GAA	TGG	CTT	TTT	GTT	GAT	GGA	GGA	TAT	ACT	CAT	TTA	GAT	AGA	GCT

257	258	259
L	V	-
CTC	GTC	TGA
TTA	GTT	TAA

FIG S3 *Burkholderia multivorans* FabG. The sequence of the original (black) and codon-optimized (blue) *B. multivorans fabG,* and protein sequence (red).

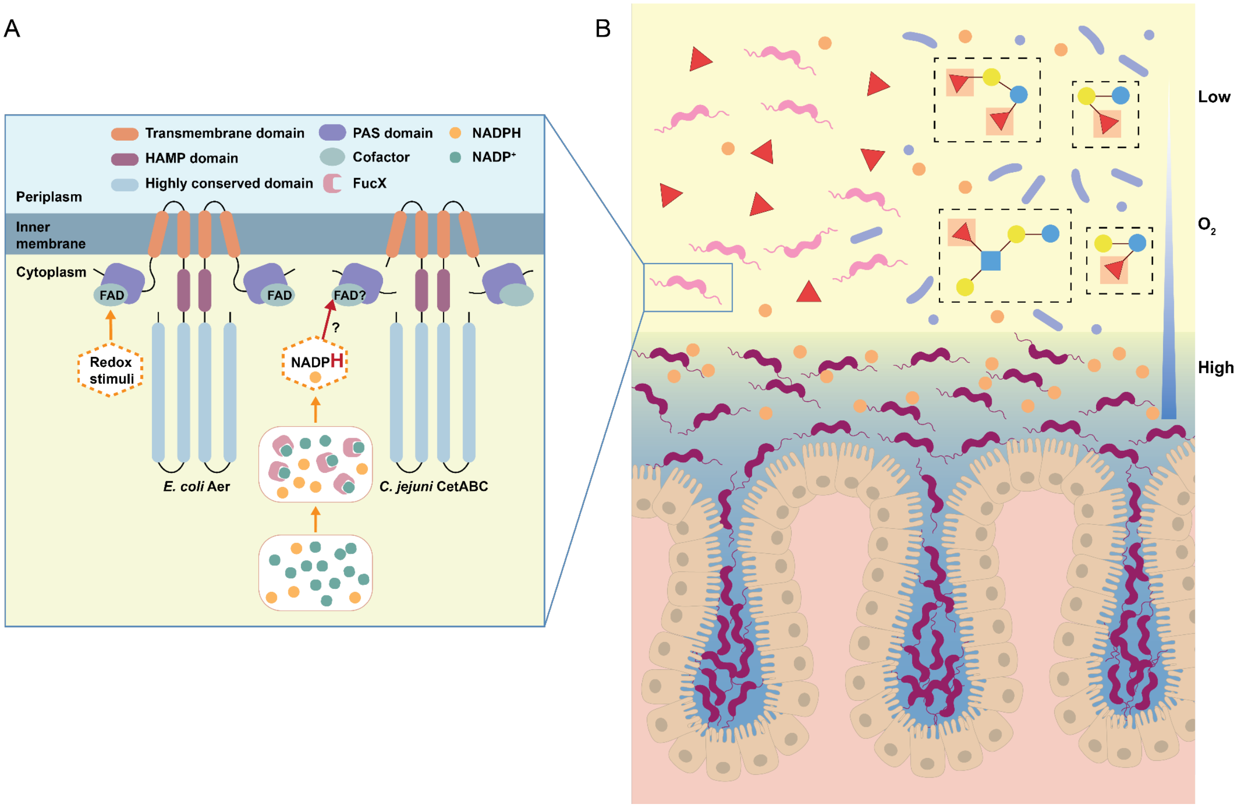

FIG S4 Comparison of the *E. coli* Aer system with the *C. jejuni* CetABC components demonstrating how colonization could be impacted by L-fucose sensing. (A) *E. coli* Aer is a membrane-embedded protein with Per-Arnt-Sim (PAS) and histidine kinase, adenylate cyclase, methyl accepting protein and phosphatase (HAMP) domains. The PAS domain binds to the flavin adenine dinucleotide (FAD) cofactor which causes protein conformational changes when FAD is reduced. These redox changes in the cell subsequently result in changes in flagellar rotation. In *C. jejuni*, FucX is expressed and binds with free $NADP^+$ in the cell in the presence of L-fucose, which increases the relative concentration of NADPH allowing for the reduction of the predicted cofactor FAD bound by CetB or CetC. This process consequently triggers flagellar rotation. (B) In the gut of breastfed infants, intestinal commensals secrete fucosidases to release L-fucose (red

triangles) from human milk oligosaccharides (HMOs); L-fucose-metabolizing *C. jejuni* move toward L-fucose and are then expelled from the gut, while non-L-fucose-metabolizing *C. jejuni* swim toward the mucin layer (darker blue) and epithelial cells along the oxygen (O_2) gradient. We hypothesize that this process is in part regulated by L-fucose chemotaxis.

References

1. Parkhill J, Wren BW, Mungall K, Ketley JM, Churcher C, Basham D, Chillingworth T, Davies RM, Feltwell T, Holroyd S, Jagels K, Karlyshev AV, Moule S, Pallen MJ, Penn CW, Quail MA, Rajandream MA, Rutherford KM, van Vliet AH, Whitehead S, Barrell BG. 2000. The genome sequence of the food-borne pathogen *Campylobacter jejuni* reveals hypervariable sequences. Nature 403:665-8.
2. Korlath JA, Osterholm MT, Judy LA, Forfang JC, Robinson RA. 1985. A point-source outbreak of campylobacteriosis associated with consumption of raw milk. J Infect Dis 152:592-6.
3. Dwivedi R, Nothaft H, Garber J, Xin Kin L, Stahl M, Flint A, van Vliet AH, Stintzi A, Szymanski CM. 2016. L-fucose influences chemotaxis and biofilm formation in *Campylobacter jejuni*. Mol Microbiol 101:575-89.
4. Garber JM, Nothaft H, Pluvinage B, Stahl M, Bian X, Porfirio S, Enriquez A, Butcher J, Huang H, Glushka J, Line E, Gerlt JA, Azadi P, Stintzi A, Boraston AB, Szymanski CM. 2020. The gastrointestinal pathogen *Campylobacter jejuni* metabolizes sugars with potential help from commensal *Bacteroides vulgatus*. Commun Biol 3:2.
5. Palyada K, Sun YQ, Flint A, Butcher J, Naikare H, Stintzi A. 2009. Characterization of the oxidative stress stimulon and PerR regulon of *Campylobacter jejuni*. BMC Genomics 10:481.
6. Blattner FR, Plunkett G, 3rd, Bloch CA, Perna NT, Burland V, Riley M, Collado-Vides J, Glasner JD, Rode CK, Mayhew GF, Gregor J, Davis NW, Kirkpatrick HA, Goeden MA, Rose DJ, Mau B, Shao Y. 1997. The complete genome sequence of *Escherichia coli* K-12. Science 277:1453-62.
7. Parkinson JS, Houts SE. 1982. Isolation and behavior of *Escherichia coli* deletion mutants lacking chemotaxis functions. J Bacteriol 151:106-13.
8. Repik A, Rebbapragada A, Johnson MS, Haznedar JO, Zhulin IB, Taylor BL. 2000. PAS domain residues involved in signal transduction by the Aer redox sensor of *Escherichia coli*. Mol Microbiol 36:806-16.
9. Yu HS, Saw JH, Hou S, Larsen RW, Watts KJ, Johnson MS, Zimmer MA, Ordal GW, Taylor BL, Alam M. 2002. Aerotactic responses in bacteria to photoreleased oxygen. FEMS Microbiol Lett 217:237-42.
10. Campbell AJ, Watts KJ, Johnson MS, Taylor BL. 2011. Role of the F1 region in the *Escherichia coli* aerotaxis receptor Aer. J Bacteriol 193:358-66.
11. Muraoka WT, Zhang Q. 2011. Phenotypic and genotypic evidence for L-fucose utilization by *Campylobacter jejuni*. J Bacteriol 193:1065-75.
12. Lefebre MD, Valvano MA. 2002. Construction and evaluation of plasmid vectors optimized for constitutive and regulated gene expression in *Burkholderia cepacia* complex isolates. Appl Environ Microbiol 68:5956-64.
13. Hendrixson DR, DiRita VJ. 2004. Identification of *Campylobacter jejuni* genes involved in commensal colonization of the chick gastrointestinal tract. Mol Microbiol 52:471-84.
